# The Potential Contribution of BRCA Mutations to Early Onset and Familial Breast Cancer in Uzbekistan

**DOI:** 10.5195/cajgh.2016.228

**Published:** 2016-12-21

**Authors:** Abdulla Abdikhakimov, Mukaddas Tukhtaboeva, Bakhtiyar Adilov, Shahlo Turdikulova

**Affiliations:** 1 Tashkent Institute of Postgraduate Education, Tashkent Regional Oncological Dispensary;; 2 Center for High Technologies Academy of Sciences Republic of Uzbekistan

**Keywords:** BRCA1, BRCA2, Founder mutation, Real-time PCR, Breast cancer, Uzbek population

## Abstract

**Introuduction:**

Breast cancer is the most common malignancy in women and affects approximately 1 out of 8 females in the US. Risk of developing breast cancer is strongly influenced by genetic factors. Germ-line mutations in BRCA1 and BRCA2 genes are associated with 5–10% of breast cancer incidence. To reduce the risk of developing cancer and to increase the likelihood of early detection, carriers of BRCA1 or BRCA2 mutations are offered surveillance programs and effective preventive medical interventions. Identification of founder mutations of BRCA1/2 in high risk communities can have a significant impact on the management of hereditary cancer at the level of the national healthcare systems, making genetic testing more affordable and cost-effective. BRCA1 and BRCA2 mutations in breast cancer patients have not been characterized in the Uzbek population. This pilot study aimed to investigate the contribution of BRCA1 and BRCA2 mutation to early onset and familial cases of breast cancer in Uzbekistan.

**Methods:**

A total of 67 patients with breast cancer and 103 age-matched disease free controls were included in this study. Utilizing SYBR Green based real-time allele-specific PCR, we have analyzed DNA samples of patients with breast cancer and disease free controls to identify the following BRCA1 and BRCA2 mutations: BRCA1 5382insC, BRCA1 4153delA, BRCA1 185delAG, BRCA1 300T>G, BRCA2 6174delT.

**Results:**

Three unrelated samples (4.5%) were found to be positive for the heterozygous 5382insCBRCA1 mutation, representing a possible founder mutation in the Uzbek population, supporting the need for larger studies examining the contribution of this mutation to breast cancer incidence in Uzbekistan. We did not find BRCA1 4153delA, BRCA1 185delAG, BRCA1 300T>G, and BRCA2 6174delT mutations.

**Conclusion:**

This preliminary evidence suggests a potential contribution of BRCA1 5382insC mutation to breast cancer development in Uzbek population. Taking into account a high disease penetrance in carriers of BRCA1 mutation, it seems reasonable to recommend inclusion of the 5382insC mutation test in future research on the development of screening programs for breast cancer prevention in Uzbekistan.

Breast cancer is one of the most commonly diagnosed malignancies around the world with the incidence potentially rising in Asia due to an increasingly westernized lifestyle.^1^ The average lifetime risk of breast cancer in women living in the US is 12.3% (1/8)^2^, and it is the most prevalent cancer in women other than skin cancer.^3^ While lifestyle factors such as obesity and reproductive factors play an important role in breast cancer development, in a small percentage of families the risk of developing breast cancer is strongly influenced by hereditary factors. The most significant and well-characterized genetic risk factors for breast cancer published to date are germline mutations of the BRCA1 and BRCA2 genes.^4^ In the general population, about 5–10% of all breast cancer cases are linked to the mutations in these genes, which can explain around half of the cases of breast cancer aggregation in some families.^5^,^6^ It is estimated that the total frequency of BRCA1 and BRCA2 mutation carriers in Europe is around 0.2% (1/500), while the prevalence of BRCA1 mutation carriers is approximately 0.13% (1/800)^7^; however, it can vary widely among different countries and ethnic groups due to the founder effect.^8^

According to the data from the Republican Oncology Research Center of the Ministry of Health of the Republic of Uzbekistan, 2273 new cases of breast cancer were diagnosed in Uzbekistan in 2010, which is 784 cases higher than in 2001 (data not published). These numbers demonstrate that the number of reported cases of breast cancer in Uzbekistan is potentially increasing. It is possible to approximate that the number of patients with breast cancer diagnosis increased by an average of 87 people per year in this time period. The rate of increase of breast cancer within a 10–year period was 37.3%.^9^ Inherited BRCA mutations are associated with high disease penetrance. Women carrying a BRCA1 mutation have a lifetime risk of 65–80% of developing breast cancer, while BRCA2 mutation carriers have a lifetime risk of 45–85%.^10^ BRCA1 and BRCA2 are tumor suppressor genes that encode large proteins of 1,863 and 3,418 amino acids, respectively.^11^ Previous research demonstrated that BRCA proteins are key regulators of important cellular processes such as DNA repair, transcription, as well as cell cycle and apoptosis in response to DNA damage.^12^

The identification of BRCA1 and BRCA2 mutation carriers is of great clinical significance, since management protocols for mutation carriers are becoming well established^13^–^15^ and effective preventive medical interventions exist.^16^,^17^ Once a mutation is identified in a given family, oncogenetic tests can be applied to all members of the affected family to identify carriers.^18^ Moreover, molecular genetic testing is becoming an important tool in predicting drug response, as new targeted therapeutic agents, such as poly(ADP-ribose) inhibitors^19^,^20^, emerge and chemosensitivity to platinum-based therapy has been reported.^21^ Improved knowledge of the genetic make-up of populations where breast cancer is increasing is very important for the development of an effective screening strategy.

Previous research demonstrated that there is a wide spectrum of mutations in the affected population.^5^ This fact makes identification of BRCA carriers difficult. A full analysis of the BRCA1 and BRCA2 genes includes not only the complete sequencing of all the coding regions of the genes, but also the detection of mutations by the multiple ligation dependent probe amplification method. In the mid-1990s, it was discovered that some populations are characterized by a pronounced “founder effect” - the predominance of the so-called recurring mutations in BRCA1 and BRCA2. For example, in Ashkenazi Jews, virtually all the mutations in BRCA1 and BRCA2 genes are related to 185delAG, 5382insC, and 6174delT.^22^ Elucidation of founder mutations of BRCA1/2 genes can have a significant impact on the management of families with hereditary cancer at the level of the national healthcare system, ultimately making genetic testing more affordable and cost-effective. To date, BRCA1 and BRCA2 mutations in breast cancer patients have not been characterized in the Uzbek population. The purpose of this pilot study is to investigate the contribution of common BRCA1 and BRCA2 mutations to early onset and familial cases of breast cancer in Uzbekistan.

## Methods

### Participants

This study included 67 Uzbek women who (1) had at least one first- or second-degree relative with breast and/or ovarian cancer, regardless of age; (2) were less than 35 years of age at diagnosis; (3) had bilateral breast cancer; (4) had triple negative (TN) or medullary type pathology; (5) had at least one relative with cancers other than breast and ovarian cancer such as stomach and prostate that are known to be related to BRCA mutations. A standard epidemiological questionnaire, including a detailed family history, was administered to all patients after obtaining informed consent. Medical information, including pathology records, was retrieved from the patients’ medical records. Information collectected from the epidemiological questionnaires included age at breast cancer diagnosis, history of other primary cancers, and a family history of breast, ovarian, and other cancers in first, second, and third degree relatives. In addition, the questionnaire included information about the usage of alcohol, tobacco, contraceptives (pills, patches, or injections), hormone replacement therapy, and prior use of infertility medications. Reproductive history (including history of breastfeeding), as well as menopausal status, was recorded for each patient. Age of patients ranged from 27 to 77 years. The median age was 43 years. Eighty-six percent of the patients were living in the Tashkent district (rural area located in the northeastern part of Uzbekistan, between the Syr Darya River and the Tien Shan Mountains) and the remaining 16% of the patients were residents of the Tashkent city. All women underwent cancer treatment in the Tashkent Regional Oncological Dispensary, Tashkent, Uzbekistan. Pathological diagnosis of breast cancer was made according to the World Health Organization (WHO) classification and the tumor–node–metastases (TNM) staging system.

Age-matched disease free control group (n=103) was identified through the clinical specialists participating in the study and included women undergoing regular prophylactic breast examinations in the Tashkent Regional Oncological Dispensary, Tashkent, Uzbekistan. They were defined as “disease free” based on the results of their last breast examinations conducted by clinicians with expertise in breast cancer. Controls were also screened for family history of breast and ovarian cancer and were determined to be free of family history of these conditions. Women in the control group were age-matched to cases and resided in Tashkent city and Tashkent Region.

All study participants signed informed consents before participating in this research. The study was conducted according to the standards of the National Ethic Committee of Uzbekistan, developed in accordance with the World Medical Association’s Declaration of Helsinki “Ethical Principles For Medical Research Involving Human Subjects” with amendments.^23^

### Laboratory methods

Blood samples (2 ml) were drawn from an antecubital vein using vacutainers containing sodium citrate, and stored at −20°C for further analysis.

Genomic DNA was extracted from peripheral blood leucocytes using DNA extraction kit Diatom™ DNA Prep 200 (“IsoGen Laboratory”, Moscow, Russia). Detection of 5 common mutations (BRCA1 5382insC, BRCA14153delA, BRCA1 185delAG, BRCA1 300T>G, BRCA2 6174delT) was performed by SYBR Green based real-time allele-specific PCR.^24^,^25^

These five common mutations were chosen based on their widespread pattern of occurrence in the world.^25^–^29^ The PCR with allele-specific primers (Table 1) was performed in a 25 µl reaction mixture containing 9.3 µl of ddH2O, 1,5 µl 10xPCR buffer containing the intercalating dye SYBR Green I, 1,5 µl 25 mM MgCl_2_, 0.3 µl 2.5 mM dNTP Mix, 0,25 µl (10pkmol/µl) of each oligonucleotide primer, 0.1 ul (0.5 units.) “hot-start” Taq-polymerase and 1.8 µl of DNA.PCR amplification was carried out in Applied Biosystems 7500 Real-Time PCR System.

The PCR conditions were optimized for each mutation and were as follows: BRCA1 5382insC (95 °C for 5 min, and then 45 cycles of 95 °C for 20 s, 55 °C for 45 sec, and 72 °C for 45 sec), BRCA1 4153delA (95 °C for 5 min, followed by 42 cycles of 95 °C for 15 sec, 63°C for 30 sec, and 72 °C for 40sec), BRCA1 185delAG (95 °C for 5 min, and then 40 cycles of 95 °C for 20 sec, 67°C for 30 sec, and 72 °C for 40 sec), BRCA1 300T>G (95 °C for 5 min, and then 45 cycles of 95 °C for 20 s, 50°C for 30 sec, and 72 °C for 40 sec) and BRCA2 6174delT (95 °C for 5 min, followed by 45 cycles of 95 °C for 10 sec, 65°C for 30 sec, and 72 °C for 45 sec).

Negative control DNA was extracted from a patient who was confirmed not to have BRCA mutations. Positive control DNA was extracted from blood obtained from a confirmed BRCA 5382insC mutation carrier. Both control DNAs were confirmed by direct DNA sequencing. Negative control, positive control, and blank tube without DNA (reagent contamination control) were included in each run.

### Statistical analysis

Descriptive statistics were done as the first step of the statistical analysis. Associations of mutations with breast cancer were evaluated by Pearson’s χ2 test under dominant model of inheritance, followed by risk assessment using odds ratio and 95% confidence interval (CI) computation. All statistical analyses were performed using STATA software version 12.0 for Windows (Stata Corporation, USA). A p-value <0.05 (two-sided) was considered statistically significant.

## Results

**Figure. 1:**
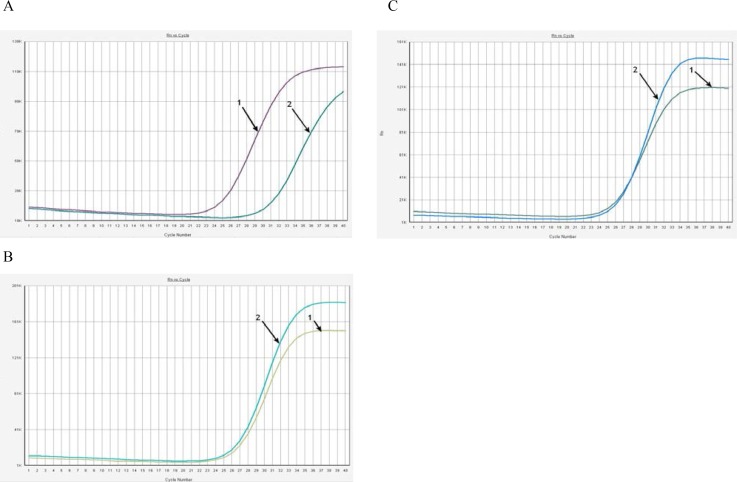
**Allele-specific PCR detection of BRCA1 5382insC mutation.**
**Negative control DNA(wild type) DNA samples show clear difference between cycle thresholds (delta Ct) of amplification of wild-type(1) and mutated alleles(2) upon real-time PCR analysis****Positive control DNA– 5382insC heterozygote mutation is characterized by simultaneous amplification of both wild-type and mutated alleles.****5382insC heterozygote mutation in patient №22**

Among 67 patients, we detected 3 cases (4.5%) with the 5382insC BRCA1 mutation (Fig. 1). Among these three patients, 1 patient had multiple primary breast cancers (multiple lesions in one breast) and 2 patients had a family history of breast cancer. In the investigated group of patients, 4 other mutations (BRCA1 4153delA, BRCA1 185delAG, BRCA1 300T>G and BRCA2 6174delT) were not found. None of the mutations were found in the control group. Comparative analysis of resulting genotypes between patients and controls showed a significant association between 5382insc mutation and breast cancer (p=0.03, Pearson’s χ2 test, dominant model of inheritance).

## Discussion

To our knowledge, this is one of the first pilot studies to explore the prevalence of BRCA mutations in women affected by breast cancer in Uzbekistan in comparison with disease free controls. The occurrence of the same 5382insC BRCA1 mutation in the 3 unrelated patients in Tashkent, Uzbekistan, may imply that this recurrent mutation originated from a common ancestor (founder mutation) and may need to be further investigated in Uzbekistan, and possibly Central Asia. The BRCA1 mutation 5382insC was originally described as a founder mutation in the Ashkenazi Jewish population. This mutation has also been detected in 13 different population groups: Russian, Latvian, Ukrainian, Czech, Slovak, Polish, Danish, Dutch, French, German, Italian, Greek, Brazilian, Turkish, and Iranian.^30^,^31^ Previously published microsatellite marker study by Hamel indicated that the 5382insC mutation of BRCA1 gene originated about 1,800 years in northern Europe and later spread to the many different populations.^30^ The presence of BRCA1 5382insC mutations in Uzbekistan is consistent with the historical, archaeological, and genetic evidence of the “hybrid zone” ethnic scenario, which postulate several waves of migration of western Caucasoid peoples followed by their integration and hybridization with East Asian people.^32^,^33^ Haplotype analysis would help to determine if 5382insC mutation found in our cohort was ancestrally related to the corresponding mutation found in other populations.^22^,^34^ It should be noted that it is quite possible that BRCA1 5382insC mutations found in **U**zbek women may have its origins in a Jewish founder.

The exact number of women in Uzbekistan with Ashkenazi Jewish background is difficult to calculate. The first Ashkenazim who came to Central Asia were merchants of the ancient Silk Road and there is evidence that the historical exchange between Ashkenazim and the Far East was not confined to cultural alone, but also extended to the sharing of genes.^35^ Large numbers of Jewish migrants moved to the Central Asian region during the Second World War when Ashkenazi Jews from Nazi-occupied countries and the Soviet Union were evacuated to Uzbekistan, but many Jewish people moved out of Central Asia after the break down of Soviet Union in 1991.

It should be noted there are several limitations of this study. First, selection of high-risk breast cancer patients in Tashkent and surrounding regions may not have accurately reflected the true prevalence of the BRCA1mutation in the geographically and ethnically diverse population of Uzbekistan. A large-scale multiregional study is needed to evaluate the prevalence of BRCA mutations on a country-wide scale. The small sample size of this study is another limitation, which we plan to address in our future work. Since the annual incidence of breast cancers in Uzbekistan in 2010 was 2273 people, the 67 women with breast cancer represent 3% of total annual incidence of breast cancers in Uzbekistan.

Despite the small sample size and detecting only 3 cases with the BRCA1 5382insC mutation, the present and future contribution s of this mutation to the incidence of breast cancer in Uzbekistan can be significant due to the so-called “founder” effect. The culture of Uzbekistan, which supports early marriage, early reproduction, and large family size, generally supports the spread of “founder” mutations. Thus, even in cases of early onset breast cancer (≤35 years old), Uzbek women with the BRCA1 mutation would have already had children; therefore, they would have transmitted the mutation to the next generation.

In discussing the importance of testing the offspring of mutation carriers identified in this study and in future research and clinical programs, it is necessary to emphasize the need to test the female as well as the male offspring. Oncogenetic testing of male members of the family is reasonable, not only because they are might be carriers of BRCA mutations and transmit the mutations to their future granddaughters, but also because men who carry the mutated BRCA1 gene have a four times greater chance of developing prostate cancer than other males.^36^ Knowledge about the mutation status for males may potentially have an impact on the screening and treatment procedures with regard to prostate cancer. It should also be noted that we have studied only a small portion of possible mutations (5382insC, 4153delA, 185delAG and 300T> G in the BRCA1 gene, and 6174delT in the BRCA2 gene) in women with breast cancer in Uzbekistan. Therefore, sequencing all exons of BRCA1 and BRCA2 genes is needed to evaluate the full spectrum of BRCA1 mutations in Uzbekistan.

## Conclusion

These pilot results suggest that there may be a potential contribution of BRCA1 5382insC mutation to breast cancer development in the Uzbek population. Women with confirmed mutations in the BRCA1 gene should be referred for a comprehensive medical examination for the early detection of breast and ovarian cancers or recieve preventive medical interventions. Diagnosis of genetic predispositions for the development of breast cancer is extremely important, as this knowledge will help to better prepare healthcare systems to organize programs for the prevention of adverse health outcomes associated with these mutations. It is very important to continue research in this area, as more data are needed on 5382insC mutation in women with breast cancer in Uzbekistan.

## Figures and Tables

**Table 1.** Oligonucleotide primers used in the study

Mutation	Primer	Sequence
BRCA1 5382insC	Common	5′-AGAACCTGTGTGAAAGTATCTAGCACTG-3′
	wt	5′-AAGCGAGCAAGAGAATTCCAG-3′
	mut	5′-AGCGAGCAAGAGAATTCCCA-3′
BRCA1 4153delA	Common	5′-GACTGCAAATACAAACACCCA-3′
	wt	5′-AGCCCGTTCCTCTTTCTTC-3′
	mut	5′-AGCCCGTTCCTCTTTCTCA-3′
BRCA1 185delAG	Common	5′-CAGTTAAGGAAATCAGCAATTACAATAGC-3′
	wt	5′-GCTATGCAGAAAATCTTAGAGTGTCC-3′
	mut	5′-ATGCTATGCAGAAAATCTTAGTGTCC-3′
BRCA1 300T>G	Common	5′-ATTATCTTTTCATGGCTATTTG-3′
	wt	5′-TATATCATTCTTACATAAAGGAA-3′
	mut	5′-TATATCATTCTTACATAAAGGAC-3′
BRCA2 6174delT	common	5′-CATAACCAAAATATGTCTGGATTGGAG-3′
	wt	5′-CTGATACCTGGACAGATTTTCCAC-3′
	mut	5′-CCTGGACAGATTTTCCCTTGC-3′

**Table 2.** The frequency of BRCA1 and BRCA2 mutations in patients with breast cancer and control group

**gene**	**mutation**	**Breast cancer patients**	**Control group (n=103)**
*BRCA1*	5382insC	3(4.5%)	0(0%)
	4153delA	0(0%)	0(0%)
	185delAG	0(0%)	0(0%)
	300T>G	0(0%)	0(0%)
*BRCA2*	6174delT	0(0%)	0(0%)

**Table 3.** Association between 5382insC mutation and breast cancer (χ2 test, df = 1, dominant model of inheritance).

Genotypes	Cases n = 67	Controls n = 103	χ2	p	value	OR 95% CI
wt/wt	0.955	1.000	4.69	0.03	0.09	0.00 – 1.75
*wt/5382insC+* *5382insC/5382insC*	0.045	1.000			11.23	0.57 – 221.04
